# Distinct Associations of Threat and Deprivation With Changes in Affective Control during Adolescence: A Longitudinal Population‐Based Study

**DOI:** 10.1111/desc.70264

**Published:** 2026-07-30

**Authors:** Sjur S. Sætren, Tore Tjora, Christian K. Tamnes, Lia Ferchman, Gertrud Sofie Hafstad, Else‐Marie Augusti

**Affiliations:** ^1^ Department for Child and Adolescent Research Norwegian Centre for Violence and Traumatic Stress Studies Oslo Norway; ^2^ Institute of Social Studies, Faculty of Social Sciences University of Stavanger Stavanger Norway; ^3^ TIPS Centre for Clinical Research in Psychosis Stavanger University Hospital Stavanger Norway; ^4^ Department of Psychology, PROMENTA Research Center University of Oslo Oslo Norway; ^5^ Division of Mental Health and Substance Abuse Diakonhjemmet Hospital Oslo Norway

**Keywords:** adverse childhood experiences, affective dysregulation, developmental psychology, emotional regulation, inhibitory control, psychology, threat

## Abstract

Childhood adversity has been linked to disruptions in the development of emotion regulation, yet few longitudinal studies have examined whether distinct dimensions of adversity are differentially associated with changes in affective inhibitory control across adolescence. This study examined whether threat and deprivation experiences differentially predicted changes in affective inhibitory control across early to middle adolescence. Participants were 902 adolescents (51.5% girls) from the UEVO study, assessed at ages 12–14 years at Time 1 (T1) and 15–16 years at Time 2 (T2). Affective inhibitory control was measured using an online emotional go/no‐go task. Adolescents showed fewer false alarms to angry no‐go stimuli at T2 than at T1, indicating improvement in affective inhibitory control over time. Latent change score modeling further supported this pattern, showing that adolescents with poorer baseline performance demonstrated greater improvement over time (*β* = −0.66, *p* < 0.001). In the primary model, higher levels of threat predicted less improvement in affective inhibitory control (*β* = 0.09, *p* = 0.013), whereas deprivation was not significantly associated with change (*β* = −0.05, *p* = 0.073). Neither threat nor deprivation predicted change in false alarms to neutral faces. In sensitivity analyses adjusting for gender, age, socioeconomic status (SES), and caregiver risk, threat remained a significant predictor of less improvement (*β* = 0.12, *p* = 0.004), whereas deprivation emerged as a small predictor of greater improvement (*β* = −0.06, *p* = 0.042). Post‐hoc multigroup analyses provided no evidence that the associations between adversity and change differed by gender. These findings suggest that threat and deprivation show partly distinct associations with affective inhibitory control, with threat‐related adversity emerging as the more consistent predictor of reduced improvement across early to middle adolescence.

## Summary

- This longitudinal study examined whether threat and deprivation predicted change in affective inhibitory control during adolescence.
- Adolescents showed overall improvement in affective inhibitory control from early to mid‐adolescence.
- Threat‐related adversity predicted reduced improvement in affective inhibitory control, whereas deprivation showed no consistent association.
- Findings support dimension‐specific links between childhood adversity and affective inhibitory control development.

## 1 Introduction

Childhood adversity is a prevalent global phenomenon (Madigan et al. 2024) and a profound risk factor for mental illness across the life span (Clark et al. 2010). While the association between childhood adversity and later psychopathology is well documented, the underlying developmental mechanisms that confer vulnerability need to be further understood. One proposed pathway involves interference with the normative development of emotion regulation (Cicchetti 2016; Miu et al. 2022; Pollak 2008), a transdiagnostic component in mental health that undergoes major development during adolescence (Ahmed et al. 2015; Schweizer et al. 2020). Given the marked increase in the onset of psychopathology during this transitional stage (Solmi et al. 2022), adolescence represents a key window for examining how early adversity shapes the development of emotion regulation (Miu et al. 2022). However, progress in clarifying these pathways has been impeded by conceptual and methodological challenges in how childhood adversity is defined and measured (McLaughlin et al. 2021; Smith and Pollak 2021) and by a scarcity of longitudinal studies. Addressing these limitations may enhance our understanding of how adversity influences developmental processes and ultimately informs more targeted and effective intervention strategies (Pollak 2024).

### 1.1 Dimensional Model of Childhood Adversity

Childhood adversity encompasses a range of potentially harmful experiences that deviate from the expected childhood environment and typically require considerable efforts of adjustment from an individual (McLaughlin 2018). The dimensional model of adversity and psychopathology (DMAP; McLaughlin and Sheridan 2016; McLaughlin et al. 2014) proposes that the diverse range of adversities experienced during childhood can be placed along dimensions of exposures that share common features. Two core dimensions presented in the model are threat and deprivation. The threat dimension encompasses experiences of actual or potential harm, including exposures such as physical, psychological, or sexual abuse, as well as witnessing violence directed toward or occurring between caregivers. In contrast, the deprivation dimension reflects the absence or insufficiency of critical inputs from the caregiving environment, such as physical and socio‐emotional neglect, leading to diminished biopsychosocial stimulation essential for healthy development (McLaughlin and Sheridan 2016). In the original work introducing the DMAP model, measures of deprivation included indices of socioeconomic status (SES; McLaughlin and Sheridan 2016; McLaughlin et al. 2014). However, recent work emphasizes that low SES is not a direct indicator of deprivation, but may increase the likelihood of reduced access to expected caregiving inputs (Berman et al. 2022; Miller et al. 2018). Similarly, other caregiver‐related adversities (e.g., parental substance abuse, mental illness) are not themselves direct indicators of either threat or deprivation, but are associated with an increased likelihood of both deprivation and threat‐related exposures (Wallimann et al. 2025).

Both theory‐driven and data‐driven analyses of the DMAP models support the model across samples of children and adolescents (Schäfer et al. 2023; Sheridan et al. 2020). The DMAP model furthermore suggests that threat and deprivation may influence specific developmental mechanisms differently. Threat‐related exposure is hypothesized to influence emotion processing systems, promoting heightened vigilance and reactivity to facilitate rapid detection of danger. Deprivation on the other hand, is thought to disrupt or attenuate the development of higher‐order cognitive functions, including cognitive control processes (McLaughlin et al. 2014). Although growing evidence supports this distinction (e.g., Machlin et al. 2019; Miller et al. 2018; Schäfer et al. 2023; Sheridan et al. 2017), it remains less clear how threat and deprivation influence cognitive control when individuals are confronted with threat‐related stimuli.

### 1.2 Childhood Adversity and Affective Inhibitory Control During Adolescence

Affective inhibitory control refers to the ability to deliberately override prepotent responses in the presence of emotional stimuli and is a key component of cognitive control under emotional conditions (Miyake and Friedman 2012; Schweizer et al. 2020). Affective inhibitory control development has frequently been investigated with behavioral measures of inhibitory control in emotional context (e.g., the emotional go/no‐go task or emotional Stroop task) across age groups, and has been shown to undergo marked maturation during adolescence, with boys tending to mature later than girls (Schweizer et al. 2020; Tottenham et al. 2011). Importantly, poorer affective inhibitory control has been associated with greater mental health difficulties across age groups, but this association has been found to be strongest in young adolescents (Schweizer et al. 2020). Individual differences in affective inhibitory control have been suggested to reflect proxies for automatic emotion regulation (Tottenham et al. 2011), and also related to explicit use of emotion regulation strategies (Schweizer et al. 2020). Better affective inhibitory control has been associated with fewer ruminative tendencies, more frequent and successful reappraisal, and less reliance on maladaptive suppression strategies (Schweizer et al. 2020). Conversely, poorer affective inhibitory control is linked to greater emotion regulation difficulties and increased risk for mental health problems in adolescence (Schweizer et al. 2020). Furthermore, it may moderate the association between threat‐related adversity and maladaptive outcomes, such as internalizing symptoms and somatic complaints (Sætren et al. 2021; Sætren et al. 2023). However, it remains poorly understood whether exposure to adversity alters the normative changes in affective inhibitory control during adolescence.

Existing evidence suggests that threat and deprivation are differentially associated with affective inhibitory control, although findings are somewhat mixed (Bounoua et al. 2025; Johnson et al. 2021; Kim et al. 2023; Lambert et al. 2017; Machlin et al. 2019; Schäfer et al. 2023). Both threat‐related and deprivation‐related adversities have been associated with poorer inhibitory control, but growing evidence suggests that these associations may differ depending on whether inhibitory control is assessed in emotional or nonemotional task contexts (Kim et al. 2023; Lambert et al. 2017). Within emotional Stroop paradigms, prior evidence suggests that only threat‐related exposure has been associated with deficits in emotional conflict adaptation, whereas deprivation appears unrelated to this process (Kim et al. 2023; Lambert et al. 2017). This pattern is further supported by findings showing that the severity of emotional and physical abuse was associated with reduced emotional conflict adaptation, while cognitive control performance in nonemotional contexts remained unaffected (Kim et al. 2023). Deprivation‐related adversity has also been associated with poorer inhibitory control, but existing evidence suggests that these associations may reflect broader deficits in cognitive control rather than difficulties specific to affective inhibitory control (Johnson et al. 2021; Kim et al. 2023; Lambert et al. 2017). In line with these findings, deprivation‐related adversity has been linked more consistently to prefrontal systems supporting higher‐order cognitive control during performance on an emotional go/no‐go task, whereas threat‐related adversity appears to be more closely associated with heightened emotional reactivity involving amygdala‐dependent networks (Bounoua et al. 2025). Together, these findings suggest that threat‐related exposure may be particularly relevant for difficulties inhibiting responses to emotionally salient cues. However, previous research has not tested whether threat‐related adversity is more strongly associated with change in affective inhibitory control for threat‐related than neutral stimuli across adolescence.

Childhood adversity may shape emotion processing and regulation by increasing the salience of threat‐related signals in the environment and promoting heightened monitoring of cues that signal possible danger (Pollak 2008). Consistent with this, developmental work suggests that maltreated children show biases in the recognition and processing of negative facial expressions, particularly fear and anger, which may reflect adaptation to hostile environments but also contribute to later socioemotional difficulties (Assed et al. 2020; McLaughlin et al. 2014; Pollak 2008). Although initially adaptive, such responses may become maladaptive over time by making it harder to disengage from threat when danger is no longer present (Pollak 2008). Previous evidence suggests that threat‐related adversity specifically (i.e., physical, psychological, and sexual abuse, and witnessing domestic violence) may increase the risk of heightened responses to threat cues (Machlin et al. 2019; Murgueitio et al. 2025; Pollak 2008; Schäfer et al. 2023). In addition, some evidence suggests that individual differences in affective inhibitory control may moderate the association between threat‐related adversity (e.g., psychological abuse) and internalizing and somatic symptoms in adolescents (Sætren et al. 2021; Sætren et al. 2023). However, longitudinal studies examining how adversity may influence developmental change in affective inhibitory control are lacking, particularly with respect to whether threat and deprivation show distinct associations with such change.

### 1.3 The Present Study

In the present study, we examined the potential impact of dimensions of childhood adversity on longitudinal change in affective inhibitory control across early to middle adolescence. Based on the DMAP model (McLaughlin and Sheridan 2016; McLaughlin et al. 2014), childhood adversity was operationalized as dimensions of threat and deprivation. Affective inhibitory control was evaluated behaviorally using an online emotional go/no‐go task in which response inhibition was assessed in the presence of neutral and angry facial expressions (Hare et al. 2008). Drawing on data from a large population‐based longitudinal sample, we evaluated the extent to which experiences of threat and deprivation assessed at time 1 (T1) prospectively predicted change in affective inhibitory control across the two‐year period between T1 and time 2 (T2). We hypothesized that (H1) normative improvement in affective inhibitory control would be reflected in a reduction in false alarms to angry stimuli between T1 and T2. Based on previous research on the DMAP model (Kim et al. 2023; Lambert et al. 2017; Machlin et al. 2019; Murgueitio et al. 2025; Schäfer et al. 2023; Sheridan et al. 2017), we further hypothesized (H2) that both threat and deprivation would be associated with attenuated developmental improvement in affective inhibitory control over time, with threat showing a stronger association than deprivation.

## 2 Method

### 2.1 Participants and Study Design

This study is part of the UEVO study, an ongoing population‑based study examining childhood adversity in adolescence in Norway (Hafstad et al. 2020). To date, the UEVO study has completed three waves of data collection among adolescents aged 12–16 years: 2019 (T1; *n* = 9240), 2020 (COVID‑sample; *n* = 3564), and 2021 (T2; *n* = 3540). Participants were recruited through stratified probability sampling of schools based on school region, school size, and whether schools had an above‐ or below‐regional average proportion of students with an immigrant background. The population of 1264 public and private schools was divided into 15 strata, consisting of five regions with three groups in each region, and schools were randomly selected within each stratum in proportion to the size of the school population. Schools for both a main and a reserve sample were selected simultaneously, and schools that declined participation were replaced by schools from the reserve sample. Schools that declined participation were replaced by schools from the reserve sample. Data were collected during school hours in participating schools through an online questionnaire and task, administered in a supervised classroom setting in Norway. Although each wave was sampled independently, a subset of adolescents participated in both T1 and T2 (*n* = 1015), forming the pool of individuals eligible for longitudinal analyses. The 2020 COVID‑specific wave was not included in the present study because it did not include data on affective inhibitory control.

For the current study, we included the prospective subsample of 902 adolescents (51.5% girls) who participated in both the T1 and T2 study waves and had valid data on the study variables. Of these, 755 completed the affective inhibitory control task at T1, 721 at T2, and 574 completed it at both time points. The final analytic sample had a mean age of 13.11 years (SD = 0.33) at T1 and 15.44 years (SD = 0.51) at T2. Descriptive statistics for the analytic sample are presented in Table 1.

**TABLE 1:** Descriptive statistics.

Variables	Total *N* = 902			Girls *n* = 452 (51.5%)				Boys *n* = 426 (48.5%)			Cohen's *d*
Age T1 (Mean/SD)	881	13.11	0.33	452	13.09	0.28	426		13.13	0.36	−0.14^*^
Age T2 (Mean/SD)	889	15.44	0.51	451	15.45	0.498	424		15.43	0.52	0.05
Childhood adversity											
Threat T1 (*n*/Mean/SD)	872	0.09	0.18	448	0.11	0.20	421		0.06	0.11	0.17^*^
Psychological abuse (*n*/%)	104	11.8		57	12.6		45		10.6		
Physical abuse (*n*/%)	139	15.9		66	14.7		71		16.7		
Sexual abuse (*n*/%)	20	2.3		15	3.3		4		0.9		
Witnessing domestic violence (*n*/%)	137	15.7		75	16.7		60		14.2		
Deprivation T1 (*n*/Mean/SD)	878	0.33	0.45	4	0.32	0.46	424		0.33	0.43	0.07
Physical neglect (*n*/%)	62	7.1		34	7.5		27		6.4		
Medical neglect (*n*/%)	104	11.9		55	12.2		48		11.3		
Emotional neglect (*n*/%)	158	17.9		78	17.3		78		18.3		
Affective inhibitory control (Mean/SD)											
FA angry T1 (0–10)	755	4.59	3.00	393	4.14	2.95	359		5.09	2.98	−0.32^*^
FA angry T2 (0–10)	721	3.25	2.66	367	2.90	2.50	332		3.56	2.80	−0.25^*^
FA neutral T1 (0–10)	755	3.14	2.59	393	2.83	2.42	359		3.48	2.73	−0.24^*^
FA neutral T2 (0–10)	721	2.44	2.63	367	2.22	2.40	332		2.56	2.78	−0.13

*Note*:Gender‐specific columns do not sum to the total sample because participants with missing gender data were excluded from gender‐stratified analyses

* *p* < 0.05.

Abbreviations: FA, false alarms; *n*, sample size; SD, standard deviation; T1, time 1; T2, time 2.

### 2.2 Measures

#### 2.2.1 Childhood Adversity—Threat and Deprivation

Threat and deprivation were assessed at T1 using a set of self‐report measures of childhood adversity from the UEVO study (Hafstad et al. 2020). For a comprehensive overview of the study and its methodology, see the UEVO Cohort Profile (Hafstad et al. 2020). For a detailed overview of the items included see Table S1. The organization of adversities into the dimensions of threat and deprivation followed the recommendations by Berman et al. (2022).

The dimension of threat includes experiences involving harm or the threat of harm. In the present study, threat was measured using the reported frequency of psychological abuse (e.g., being mocked in a hurtful way or threatened by an adult), physical abuse (e.g., being hit, kicked, or pushed), sexual abuse by an adult (e.g., an adult made the child touch their private parts), and witnessing domestic violence (e.g., seeing an adult threaten or hurt someone else at home). Items were rated on a 4‐point scale ranging from 0 (never) to 3 (often), and a mean score was calculated, allowing for up to 20% missing responses.

The dimension of deprivation reflects reduced access to expected caregiving inputs, including emotional, physical, and social stimulation. In the present study, deprivation was assessed using items reflecting physical neglect (e.g., “I had to go hungry because we didn't have enough food at home”), emotional neglect (e.g., “Someone in my family made me feel valued”) and medical neglect (e.g., “I was taken to the doctor when I needed it”). Items were rated on a 5‐point scale ranging from 0 (never) to 4 (always). A mean score was then calculated, allowing for up to 20% missing responses.

#### 2.2.2 Contextual Risk Factors for Threat and Deprivation

Self‑perceived socioeconomic status (SES) and caregiver risk factors were included as contextual risk factors for direct exposure to adversity. SES was assessed with two items reflecting perceived economic strain (e.g., “I feel that my family can generally afford to buy what we need,” rated from 0 = Applies very well to 3 = Applies very poorly; and “I had to drop an activity I liked because my parents couldn't afford it,” rated 0 = Never to 3 = Often). Mean SES score was calculated. Caregiver risk factors were measured with three dichotomous items asking whether the adolescent had lived with an adult who: (1) had a problem with alcohol, (2) had a mental illness, or (3) had been in prison (No = 0; Yes = 3). A sum score (range 0–9) was calculated to represent cumulative exposure to caregiver risk.

#### 2.2.3 Affective Inhibitory Control

**FIGURE 1:**
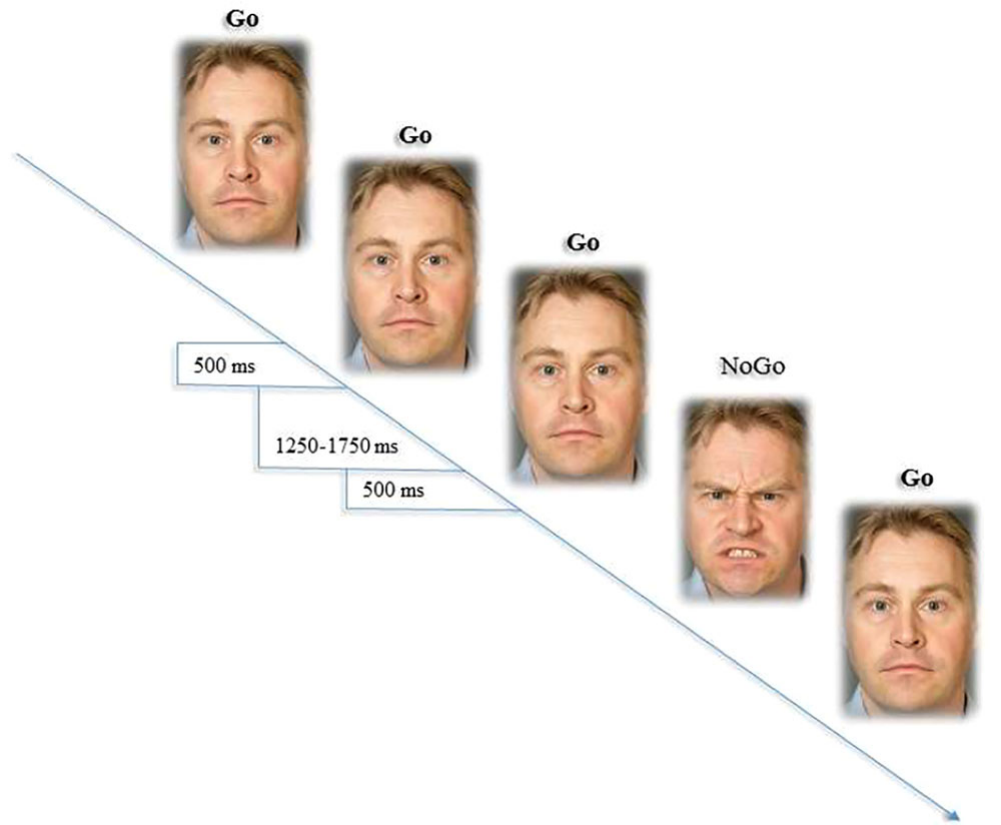
Visual illustration of stimulus presentation in the emotional go/no‐go task. *Note*. The pictures in this figure are from “The Umeå University Database of Facial Expressions: A Validation Study,” by H. Samuelsson, K. Jarnvik, H. Henningsson, J. Andersson, and P. Carlbring, 2012, *Journal of Medical Internet Research*, *14*(5), e136. Copyright [2012] by the Umeå University Database (Per Carlbring). Reprinted with permission. See the online article for the color version of this figure.

Affective inhibitory control was assessed at T1 and T2 using an emotional go/no‐go task (Hare et al. 2008), which measures participants' capacity to regulate emotional responses by requiring them to either respond or inhibit a response based on emotional stimuli (Figure 1). Participants completed two blocks of trials featuring adults with neutral and angry facial expressions as the target and nontarget stimuli. Each block consisted of 32 trials, with 22 go trials and 10 no‐go trials. The sequence of target and nontarget stimuli was pseudo‐randomized. Participants were instructed to respond as quickly as possible by pressing the spacebar on go trials and withholding their response on no‐go trials. Facial expressions were presented centrally on the screen following a fixation cross (+). A higher proportion of go trials (70/30 ratio) was used to create a bias toward automatic responses on go trials. Each stimulus was displayed for 500 ms, with a randomly varying interstimulus interval between 1250 and 1750 ms. For the analyses, false alarms to angry no‐go stimuli (FA angry) were computed at T1 and T2 and used as the primary indicator of affective inhibitory control. False alarms to neutral no‐go stimuli (FA neutral) were also calculated.

### 2.3 Statistical Analysis

Descriptive statistics for threat, deprivation, and affective inhibitory control (FA angry and FA neutral) at T1 and T2 were calculated in SPSS version 29 (George and Mallery 2024). To further characterize exposure to threat and deprivation in the sample, prevalence estimates were derived by creating binary indicators of exposure for each subtype of adversity comprising the two dimensions. Participants were coded as exposed if they endorsed a score above a predefined threshold on at least one item within each subtype. For psychological abuse and all neglect indicators, exposure was defined as a response of ≥ 2, reflecting repeated exposure. For physical violence, sexual abuse, and witnessing violence, exposure was defined as a response of ≥ 1, indicating any occurrence. Prevalence was calculated as the proportion of participants classified as exposed within each subtype.

To test whether affective inhibitory control changed from T1 to T2 (H1), we first conducted a paired‐samples *t*‐test in SPSS version 29 (George and Mallery 2024) as an initial test of mean‐level change. We then estimated a univariate latent change score (ULCS) model in the lavaan package in R (Rosseel 2012), following the recommendations provided by Kievit et al. (2018). In this model, affective inhibitory control at T2 was regressed on T1 scores with the regression coefficient fixed to 1. A latent change factor (ΔFA angry) was specified to capture residualized change from T1 to T2, allowing estimation of both mean change and interindividual variability in change. To model proportional change, the latent change factor was regressed on baseline affective inhibitory control, allowing initial levels to predict subsequent change. Accordingly, because the change factor was regressed on baseline levels, the intercept reflects the conditional expected change when baseline false alarms is zero, rather than the average raw change observed in the sample.

To test whether threat and deprivation, as dimensions of childhood adversity, predicted change in affective inhibitory control (H2), we extended the ULCS model by including threat and deprivation as simultaneous exogenous predictors of the latent change factor. This approach followed recommendations for modeling predictors of change in ULCS frameworks (Klopack and Wickrama 2020; Vecchione and Zuffianò 2024). Baseline affective inhibitory control was retained as a predictor of the latent change factor to model proportional change, and the covariance between threat and deprivation was freely estimated. This allowed us to examine whether higher levels of threat or deprivation were associated with less improvement in affective inhibitory control, reflected in a smaller decrease in false alarms to angry faces (FA angry) over time. All exogenous variables were treated as random (fixed.x = FALSE), allowing estimation of their means, variances, and covariances and enabling full information maximum likelihood (FIML) handling of missing data. Models were estimated using robust maximum likelihood, and model fit was evaluated using robust fit indices, including the CFI, TLI, RMSEA, and SRMR. To examine whether these associations were specific to affective inhibitory control in the context of angry no‐go stimuli, the same ULCS modeling approach was also applied to false alarms to neutral faces in no‐go trials.

To test the robustness of the primary model, we conducted a sensitivity analysis in which the ULCS model was reestimated adjusting for gender, age at T1, SES, and caregiver risk. Threat and deprivation, together with all covariates, were included as simultaneous predictors of the latent change factor (ΔFA angry) and of baseline affective inhibitory control at T1, allowing us to examine whether associations between threat and deprivation and change in affective inhibitory control were robust to adjustment for relevant covariates and whether adversity‐related differences were already present at baseline. Covariances among all exogenous variables were freely estimated. As in the primary analyses, exogenous variables were treated as random (fixed.x = FALSE), models were estimated using robust maximum likelihood, and missing data were handled using FIML. Model fit was evaluated using robust fit indices, including the CFI, TLI, RMSEA, and SRMR.

To explore potential gender differences in the associations of threat and deprivation with change in affective inhibitory control, we conducted post hoc multigroup latent change score analyses. We first estimated a model in which the paths from threat and deprivation to the latent change factor were freely estimated across gender and then compared it with a model in which these paths were constrained to be equal across gender. Nested model comparisons were conducted using robust chi‐square difference tests.

### 2.4 Attrition Analysis and Missing Data

To assess potential bias due to deviation in sample characteristics from the original sample at T1, we compared participants at baseline who did not take part at T2 (*n* = 8338) with those participating in the study (*n* = 902). Independent samples *t*‐tests revealed that participants lost to follow‐up reported significantly higher levels of threat exposure (*M* = 0.10, *SD* = 0.21) compared to those retained (*n* = 860*, M* = 0.07, *SD* = 0.17; *t*(1149.02) = 4.52, *p* < 0.001, *d* = 0.14). Similarly, baseline deprivation scores were higher among dropouts (*M* = 0.22, *SD* = 0.31) than among retained participants (*M* = 0.18, *SD* = 0.26; *t*(1143.01) = 3.74, *p* < 0.001, *d* = 0.12). Participants lost to follow‐up also showed significantly lower false alarm rates to angry faces at T1 (*M* = 4.32, *SD* = 2.98) compared to those retained (*M* = 4.59, *SD* = 3.00; *t*(7239) = −2.36, *p* = 0.018, *d* = −0.09), suggesting that the final sample displayed somewhat reduced affective inhibitory control at baseline. Importantly, these differences were small and likely reflect, at least in part, the age structure of the longitudinal sample. The analytic sample primarily consisted of the youngest adolescents at T1, as older adolescents from the baseline cohort were less likely to be enrolled in the schools included in later waves of data collection. Thus, lower adversity exposure and poorer affective inhibitory control in the retained sample may partly reflect younger age at baseline rather than selective attrition alone.

Missing data were compared across the full baseline sample (*N* = 9240) and the current study sample (*N* = 902). Baseline adversity measures showed moderate levels of missingness in the full sample, with 8.7% and 7.3% missing for threat and deprivation respectively, and 25.8% missing on baseline affective inhibitory control (FA angry at T1). For participants with data on both time points, missingness on these same variables were comparable (6.9% for threat and 5.5% for deprivation; 25.6% for FA Angry T1 and 29.0% for FA Angry T2). Importantly, the overall pattern and proportion of missingness was consistent across samples, suggesting that missingness was likely due to broader procedural factors (e.g., task non‐completion, use of tablet vs computer) rather than selective attrition. These patterns support the assumption that the data were missing at random.

### 2.5 Ethics

The study was approved by the Regional Committee for Medical and Health Research Ethics in Norway (Case #2018/522). In accordance with the Norwegian Act on Medical and Health Research (Helseforskningsloven 2008), adolescents were permitted to provide informed consent independently of their parents. A thorough post‐survey follow‐up protocol was implemented to ensure that any participant who experienced distress or required support after completing the survey received appropriate care.

## 3 Results

### 3.1 Descriptive and Preliminary Statistics

Descriptive statistics for the study variables are presented in Table 1. The sample consisted of 902 adolescents (51.5% girls), ranging in age from 12 to 14 years at T1 (*M* = 13.11, *SD* = 0.33) and from 15 to 16 years at T2 (*M* = 15.44, *SD* = 0.51). Prevalence estimates ranged from 2.3% to 17.9% across adversity subtypes, suggesting that individual adversity subtypes were reported at relatively low frequencies. In line with the population‐based nature of the sample, mean levels of deprivation (*M* = 0.33, *SD* = 0.45) and threat (*M* = 0.09, *SD* = 0.18) were low at T1. Boys reported slightly lower levels of threat than girls (*d* = 0.17), whereas gender differences in deprivation were minimal (*d* = 0.07). Affective inhibitory control, indexed by false alarms to angry faces, showed improvement from T1 (*M* = 4.59, *SD* = 3.00) to T2 (*M* = 3.25, *SD* = 2.66). Girls demonstrated better affective inhibitory control than boys at both time points (T1: *d* = −0.32; T2: *d* = −0.25).

### 3.2 Longitudinal Change in Affective Inhibitory Control across Early to Middle Adolescence

First, we examined group‐level changes in affective inhibitory control using a paired‐samples *t*‐test. Adolescents made significantly fewer false alarms at T2 (*M* = 3.06, *SD* = 2.58) than at T1 (*M* = 4.55, *SD* = 2.95), *t*(573) = 10.69, *p* < 0.001, *d* = 0.45, 95% CI [0.36, 0.53]. This indicated an overall reduction in false alarms to angry no‐go trials across the 2‐year period. To examine developmental change in more detail, we estimated a ULCS model (Kievit et al. 2018). The intercept of the latent change factor was significant (*b* = 2.08, *SE* = 0.18, *p* < 0.001), and there was significant variance in change (Var = 6.52, *p* < 0.001), indicating significant variability in change over the study period. Baseline affective inhibitory control strongly predicted subsequent change (β = −0.66, *p* < 0.001). The negative proportional change parameter indicated that adolescents with higher false alarm rates at T1 showed larger reductions over time, whereas those with lower initial rates showed less change. Thus, although the descriptive analyses indicated an overall reduction in false alarms, the ULCS model showed that change in affective inhibitory control was baseline‐dependent rather than uniform across participants. Because this baseline model was just‐identified, global fit indices were not interpreted.

### 3.3 Threat and Deprivation as Predictors of Change in Affective Inhibitory Control

**FIGURE 2:**
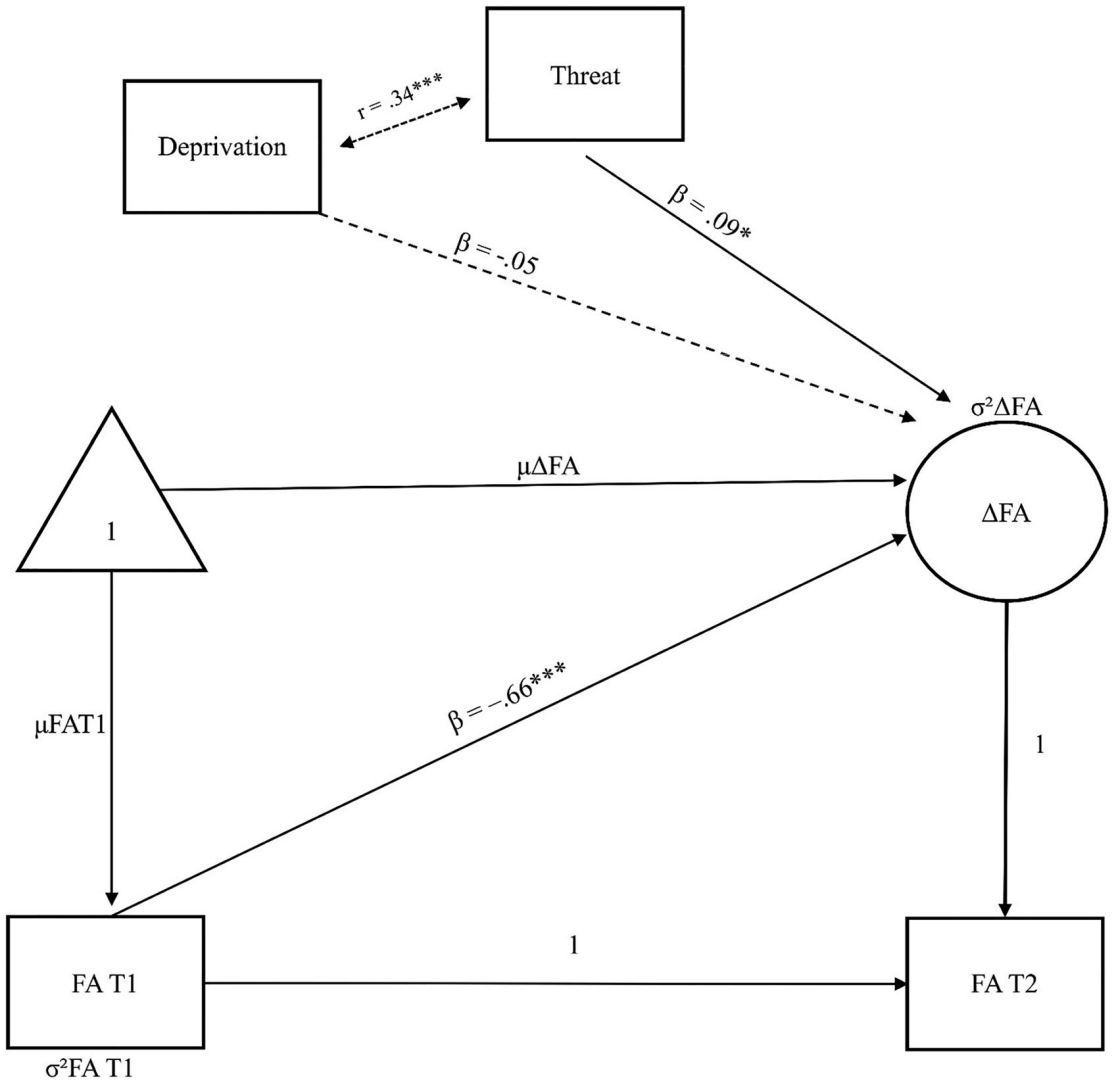
Latent change score model—Threat and deprivation predicting change in affective inhibitory control in adolescence. *Note*. FA T1 and FA T2 represent false alarms to angry faces at Time 1 and Time 2. ΔFA represents the latent change score, capturing individual differences in change in affective inhibitory control from T1 to T2. Threat and deprivation were included as simultaneous predictors of ΔFA, while baseline affective inhibitory control was retained as a predictor of change. Standardized coefficients (*β*) are shown. Dashed lines indicate non‐significant paths. * = *p* < .05, ** = *p* < .01, ** = *p* < .001.

We next extended the ULCS model by including threat and deprivation at T1 as simultaneous predictors of change in affective inhibitory control (ΔFA angry). This allowed us to examine whether higher levels of threat and deprivation were associated with less improvement in affective inhibitory control across early to middle adolescence, while accounting for baseline affective inhibitory control through the proportional change parameter. In the combined model (see Figure 2), higher levels of threat significantly predicted less improvement in affective inhibitory control (*β* = 0.09, *p* = 0.013), whereas deprivation was not a significant predictor of change (*β* = −0.05, *p* = 0.073). Baseline affective inhibitory control strongly predicted subsequent change (*β* = −0.66, *p* < 0.001). Threat and deprivation were positively correlated (*r* = 0.34, *p* < 0.001). The model showed good fit to the data (*χ* ^2^(2) = 0.99, *p* = 0.611, robust CFI = 0.998, robust TLI = 0.995, robust RMSEA = 0.013, and SRMR = 0.018).

To examine whether these associations were specific to affective inhibitory control in the context of angry no‐go stimuli, we estimated the same ULCS model using false alarms to neutral faces as the outcome. In contrast to the findings for angry faces, neither threat (*β* = 0.06, *p* = 0.117), nor deprivation (*β* = 0.03, *p* = 0.237), significantly predicted change in false alarms to neutral faces. Baseline false alarms to neutral faces strongly predicted subsequent change (*β* = −0.63, *p* < 0.001). The model showed acceptable fit to the data (*χ* ^2^(2) = 5.25, *p* = 0.072), robust CFI = 0.974, robust TLI = 0.923, robust RMSEA = 0.047, and SRMR = 0.025).

To test the robustness of the primary findings, we reestimated the ULCS model adjusting for gender, age at T1, SES, and caregiver risk. The sensitivity model showed acceptable to good fit to the data (*χ* ^2^(2) = 4.21, *p* = 0.122, robust CFI = 0.992, robust TLI = 0.890, robust RMSEA = 0.049, and SRMR = 0.013). Higher levels of threat remained a significant predictor of less improvement in affective inhibitory control (*β* = 0.12, *p* = 0.004). In contrast, deprivation emerged as a small but significant predictor increased improvement in affective inhibitory control (*β* = −0.06, *p* = 0.042). Baseline affective inhibitory control remained a strong predictor of subsequent change (*β* = −0.67, *p* < 0.001). Among the covariates, gender significantly predicted change (*β* = 0.08, *p* = 0.006), whereas age at T1, SES, and caregiver risk were not significantly associated with change (all *p*‐values > 0.05). Gender also significantly predicted baseline affective inhibitory control (*β* = 0.16, *p* < 0.001), whereas age at T1, SES, and caregiver risk were not significantly associated with baseline levels (all *p*‐values > 0.05). Because girls were coded as 0 and boys as 1, the positive gender coefficients indicate that boys made more false alarms to angry faces at T1 and showed less improvement over time compared with girls. Threat and deprivation were positively correlated (*r* = 0.34, *p* < 0.001).

### 3.4 Post‐Hoc Gender Analyses

Post‐hoc multigroup analyses were conducted to examine whether the associations between threat, deprivation, and change in affective inhibitory control differed by gender. A model in which the paths from threat and deprivation to the latent change factor were freely estimated across gender was compared with a model in which these paths were constrained to be equal across gender. Constraining these paths did not significantly worsen model fit, Δ*χ* ^2^(5) = 1.41, *p* = 0.923, providing no evidence that the associations between threat, deprivation, and change in affective inhibitory control differed by gender.

## 4 Discussion

The present study examined change in affective inhibitory control from early to middle adolescence and whether exposure to threat and deprivation, conceptualized as dimensions of childhood adversity, was associated with less improvement in this capacity over a two‐year period. Using a latent change score approach in a longitudinal population‐based sample, the findings indicated an overall reduction in false alarms to angry no‐go stimuli from T1 to T2, consistent with our hypothesis of normative improvement in affective inhibitory control across the study period. At the same time, change was not uniform across participants, as adolescents with poorer affective inhibitory control at baseline showed larger reductions in false alarms over time, whereas those with better initial performance showed less change. Regarding threat and deprivation, measures of childhood adversity in the present study showed different patterns of association with change in affective inhibitory control. Higher levels of threat at baseline were consistently associated with less improvement over time, whereas deprivation did not independently predict change in the primary model. In the comparison analysis using false alarms to neutral no‐go stimuli neither threat nor deprivation predicted change, suggesting that the observed association was specific to affective inhibitory control in the context of angry no‐go stimuli.

Consistent with our first hypothesis and prior developmental research (Schweizer et al. 2020), adolescents showed a reduction in false alarms to angry faces from T1 to T2, suggesting improvement in affective inhibitory control across the study period. Prior work has reported age‐related differences in affective inhibitory control across adolescence, including in studies comparing adolescents with children and adults (Schweizer et al. 2020; Tottenham et al. 2011). Extending this work, our longitudinal latent change score model indicated that change was strongly related to baseline performance, such that adolescents with higher levels of false alarms at T1 showed larger reductions in false alarms from T1 to T2. The magnitude of this association suggests that baseline performance was an important predictor of change over the study period. However, because the study included only two time points, this association should be interpreted cautiously rather than as strong evidence of differential developmental trajectories. It may partly reflect regression to the mean, whereby adolescents with poorer initial performance had more room to improve and were statistically more likely to show reductions over time. Thus, although the findings indicate average improvement in affective inhibitory control from T1 to T2, conclusions regarding individual differences in developmental change should remain tentative and should be further examined in longitudinal designs with more than two time points.

Moreover, our second hypothesis was partly supported, with the findings indicating that threat and deprivation were not equally associated with change in affective inhibitory control over time. Consistent with the DMAP model (McLaughlin and Sheridan 2016; McLaughlin et al. 2014), higher levels of threat were associated with less improvement in affective inhibitory control across the study period, even when deprivation was included in the same model. This pattern aligns with prior work suggesting that experiences involving harm or threat of harm may be particularly relevant for difficulties inhibiting responses in emotionally salient contexts (Kim et al. 2023; Lambert et al. 2017; Schäfer et al. 2023). One possible interpretation is that exposure to threat‐related adversity heightens the salience of threatening cues and promotes increased monitoring of signals associated with danger (Machlin et al. 2019; Schäfer et al. 2023), which may make it more difficult to disengage from emotionally salient information and override prepotent responses when threat is no longer relevant (Pollak 2008). Prior work suggests that neglect and related forms of deprivation may disrupt the broader development of emotion recognition and differentiation (Pollak 2008), rather than leading to a specific threat‐related bias (Murgueitio et al. 2025). In the present study, threat predicted reduced improvement in affective inhibitory control, whereas neither threat nor deprivation significantly predicted change in performance to neutral no‐go stimuli. This pattern is consistent with our hypothesis that threat‐related adversity may be particularly relevant for affective inhibitory control. However, cautious interpretation is warranted, as the effect estimate for threat was broadly comparable across emotional and neutral no‐go conditions, despite differences in statistical significance. Importantly, the association between threat and reduced improvement in affective inhibitory control remained evident in sensitivity analyses adjusting for gender, age, SES, and caregiver risk, suggesting that the observed pattern was not explained by broader contextual adversity. In these analyses, deprivation was associated with greater improvement in affective inhibitory control, although this association was small and should be interpreted cautiously. A similar, but nonsignificant, pattern was observed in the main model. One possible explanation is that, when threat and deprivation are modeled simultaneously, the deprivation coefficient reflects variation in deprivation that is independent of threat‐related adversity. However, given the small magnitude and inconsistency of this association across analyses, this interpretation remains speculative and requires replication. Taken together, the findings suggest that threat‐related adversity may be particularly relevant for change in affective inhibitory control.

While the present study provides novel insights into how childhood adversity may influence changes in affective inhibitory control during adolescence, several limitations should be acknowledged. First, the observational nature of the data limits causal inference. As previously discussed (Danese and Widom 2024; Danese et al. 2017), associations between adversity and neurocognitive outcomes may partly reflect preexisting vulnerabilities rather than adversity exposure per se, a possibility that cannot be ruled out given the present study design. Second, although our operationalization of deprivation focused on direct experiences of physical and socio‐emotional neglect, it did not include indicators of cognitive deprivation, such as reduced learning opportunities or limited cognitive stimulation in the home environment. As contemporary dimensional models emphasize both socio‐emotional and cognitive inputs as core components of deprivation (Berman et al. 2022; Tsai et al. 2025), our measure captures only part of this dimension. Consequently, the present findings should be interpreted with caution, as they may not fully reflect the broader range of mechanisms theorized to link deprivation with neurocognitive development, particularly those related to cognitive stimulation and higher‐order executive functioning (McLaughlin and Sheridan 2016; McLaughlin et al. 2014). Third, affective inhibitory control was measured using a single indicator, the emotional go/no‐go task with relatively few no‐go trails (Hare et al. 2008). Third, affective inhibitory control was measured using a single indicator, the emotional go/no‐go task, with relatively few no‐go trials, and administration in a classroom setting may have introduced some noise. However, schools were instructed to conduct the task under exam‐like conditions, and prior work using the same cohort (Hafstad et al. 2020) has found false‐alarm rates comparable to laboratory‐based studies using similar tasks (Tottenham et al. 2011). Fourth, although gender differences were observed in baseline levels of affective inhibitory control, post‐hoc multigroup analyses provided no evidence that the associations between threat, deprivation, and change in affective inhibitory control differed by gender. Given the exploratory and post‐hoc nature of these analyses, future studies should further examine whether gender moderates the associations between adversity dimensions and affective inhibitory control. Fifth, although differences between the final analytic sample and those not retained from baseline were small, the analytic sample reported lower levels of threat and deprivation at T1. This may have reduced variability in adversity exposure, limited generalizability, and attenuated associations between childhood adversity and change in affective inhibitory control. Finally, the study did not capture dimensions of unpredictability, timing or chronicity of adversity exposure (McLaughlin et al. 2021). This limits our ability to explore whether other important dimensions of adversity might moderate changes in affective inhibitory control.

In conclusion, our findings support the DMAP framework (McLaughlin and Sheridan 2016; McLaughlin et al. 2021), with threat‐related adversity emerging as a more consistent predictor of reduced improvement in affective inhibitory control during adolescence. Although causal inferences cannot be drawn, these findings suggest that threat‐related experiences may be particularly relevant for understanding individual differences in affective inhibitory control development. Future research should examine mechanisms linking threat exposure to affective inhibitory control and clarify how such changes are related to the development of more explicit emotion regulation strategies during adolescence.

## Author Contributions

**Sjur S. Sætren**: conceptualization, investigation, writing – original draft, methodology, writing – review and editing, formal analysis, visualization. **Tore Tjora**: conceptualization, investigation, methodology, writing – review and editing, supervision, formal analysis. **Christian K. Tamnes**: conceptualization, investigation, methodology, writing – review and editing, supervision. **Lia Ferchman**: conceptualization, investigation, methodology, writing – review and editing, supervision. **Gertrud Sofie Hafstad**: funding acquisition, project administration, writing – review and editing. **Else‐Marie Augusti**: conceptualization, investigation, writing – original draft, methodology, validation, writing – review and editing, formal analysis, supervision.

## Funding

The authors have nothing to report.

## Conflicts of Interest

The authors declare no conflicts of interest.

## Supporting information




**Supporting Information**: desc70264‐supp‐0001‐SuppMat.docx

## Data Availability

The data that support the findings of this study are available on request from the corresponding author. The data are not publicly available due to privacy or ethical restrictions.
