# Supplementary material for: Distinct Associations of Threat and Deprivation With Changes in Affective Control during Adolescence: A Longitudinal Population‐Based Study
Source: Dev Sci. 2026 Jul 30;29(5):e70264. doi: 10.1111/desc.70264 (PMC13424827; doi:10.1111/desc.70264)
Supplement: Supplementary file 1 — Supporting Information: desc70264‐supp‐0001‐SuppMat.docx [file DESC-29-e70264-s001.docx]

**Supplementary tables**

**Table 1** Threat and Deprivation Measurement

| **Threat** |  |  |
| --- | --- | --- |
| Psychological abuse | Has an adult at home ever: |  |
|  | Mocked you in a hurtful way | Never (0); Once (1); Sometimes (2); Often (3) |
|  | Said you were stupid or incapable | Never (0); Once (1); Sometimes (2); Often (3) |
|  | Threatened to leave you or send you away | Never (0); Once (1); Sometimes (2); Often (3) |
|  | Threatened to hit or hurt you | Never (0); Once (1); Sometimes (2); Often (3) |
|  | Locked you out of your home | Never (0); Once (1); Sometimes (2); Often (3) |
|  | Locked you in a basement, closet, or similar place | Never (0); Once (1); Sometimes (2); Often (3) |
|  | Threatened to harm the family pet | Never (0); Once (1); Sometimes (2); Often (3) |
|  |  |  |
| Physical abuse | Has an adult at home ever: |  |
|  | Pulled your hair or pinched you | Never (0); Once (1); Sometimes (2); Often (3) |
|  | Hit you with an open hand | Never (0); Once (1); Sometimes (2); Often (3) |
|  | Shaken or pushed you violently | Never (0); Once (1); Sometimes (2); Often (3) |
|  | Hit you with a fist or hard object | Never (0); Once (1); Sometimes (2); Often (3) |
|  | Kicked you | Never (0); Once (1); Sometimes (2); Often (3) |
|  | Beaten you up | Never (0); Once (1); Sometimes (2); Often (3) |
|  |  |  |
| Sexual abuse by adult | Has an ADULT ever: |  |
|  | Made you touch their private body parts? | Never (0); Once (1); Sometimes (2); Often (3) |
|  | Touched your private body parts? | Never (0); Once (1); Sometimes (2); Often (3) |
|  | Had (or attempted to have) sex with you (e.g., touching, oral sex, intercourse)? | Never (0); Once (1); Sometimes (2); Often (3) |
| Witnessing violence between parents | Have you seen your MOTHER experience any of the following at home? |  |
|  | Been yelled at | Never (0); Once (1); Sometimes (2); Often (3) |
|  | Been mocked | Never (0); Once (1); Sometimes (2); Often (3) |
|  | Been pushed or shaken violently | Never (0); Once (1); Sometimes (2); Often (3) |
|  | Been hit | Never (0); Once (1); Sometimes (2); Often (3) |
|  | Been beaten up | Never (0); Once (1); Sometimes (2); Often (3) |
|  | Been exposed to other violent acts | Never (0); Once (1); Sometimes (2); Often (3) |
|  | Have you seen your FATHER experience any of the following at home? |  |
|  | Been yelled at | Never (0); Once (1); Sometimes (2); Often (3) |
|  | Been mocked | Never (0); Once (1); Sometimes (2); Often (3) |
|  | Been pushed or shaken violently | Never (0); Once (1); Sometimes (2); Often (3) |
|  | Been hit | Never (0); Once (1); Sometimes (2); Often (3) |
|  | Been beaten up | Never (0); Once (1); Sometimes (2); Often (3) |
|  | Been exposed to other violent acts | Never (0); Once (1); Sometimes (2); Often (3) |
|  |  |  |
|  |  |  |
| **Deprivation** |  |  |
|  | Thinking about your life, have you experienced any of the following? |  |
| Physical Negelct | I had to go hungry (because we didn’t have enough food at home) | Never (0); Rarely (1); Sometimes (2); Often (3); Very often/Always (4) |
| Physical Neglect | I had to wear dirty clothes | Never (0); Rarely (1); Sometimes (2); Often (3); Very often/Always (4) |
| Physical Neglect | My parents were so affected by alcohol, drugs, or pills that they couldn't take care of me | Never (0); Rarely (1); Sometimes (2); Often (3); Very often/Always (4) |
| Medical neglect | I was taken to the doctor when I needed it **R** | Never (0); Rarely (1); Sometimes (2); Often (3); Very often/Always (4) |
| Emotional neglect | Someone in my family made me feel valued **R** | Never (0); Rarely (1); Sometimes (2); Often (3); Very often/Always (4) |
| Emotional neglect | I felt that someone loved me **R** | Never (0); Rarely (1); Sometimes (2); Often (3); Very often/Always (4) |

**R** = reverse-coded response scale.
